# Patterns of Semantic Memory Impairment in Mild Cognitive Impairment

**DOI:** 10.1155/2008/859657

**Published:** 2008-04-11

**Authors:** Sven Joubert, Olivier Felician, Emmanuel J. Barbeau, Mira Didic, Michel Poncet, Mathieu Ceccaldi

**Affiliations:** ^1^Centre de RechercheInstitut Universitaire de Gériatrie de MontréalCanada; ^2^Département de Psychologie et CERNECUniversité de MontréalCanada; ^3^AP-HM Timone and Laboratoire de Neurophysiologie et de NeuropsychologieUniversité de la MéditerranéeINSERM U751MarseilleFrance; ^4^Centre de Recherche Cerveau et CognitionUniversité Paul Sabatier Toulouse 3ToulouseFrance

## Abstract

Although the semantic memory impairment has been largely documented in Alzheimer's disease, little is known about semantic memory in the preclinical phase of the disease (Mild Cognitive Impairment). The purpose of this study was to document the nature of semantic breakdown using a battery of tests assessing different aspects of conceptual knowledge: knowledge about common objects, famous people and famous public events. Results indicate that all domains of semantic memory were impaired in MCI individuals but knowledge about famous people and famous events was affected to a greater extent than knowledge about objects. This pattern of results suggests that conceptual entities with distinctive and unique properties may be more prone to semantic breakdown in MCI. In summary, results of this study support the view that genuine semantic deficits are present in MCI. It could be useful to investigate the etiological outcome of patients failing or succeeding at such tests.

## 1. Introduction

Semantic memory is the component of declarative memory that refers to general world knowledge acquired over a life time and shared by a same cultural group [19]. Although the semantic memory impairment in Alzheimer's disease (AD) has been widely documented [5,12,13], few studies have investigated the longitudinal dimension of semantic deficits over the course of the disease [10] and surprisingly very little is known about semantic memory in the preclinical phase of the disease, generally referred to as Mild Cognitive Impairment (MCI) [17]. Episodic memory impairments are considered to be the hallmark and the earliest cognitive manifestation of AD. Consequently episodic memory has remained the center of interest of a majority of studies investigating patterns of memory breakdown in the very early stage of the disease [13].

One of the first studies to document semantic impairments in a group of minimal AD patients (MCI) was carried out by Hodges and Patterson [12]. The pattern was heterogeneous, however, with some patients showing deficits on all subtests, others only on certain subtests while a minority did not present with any deficits. Recently, a small number of studies have also suggested that MCI patients present with semantic memory impairments. For instance, Adlam et al. [1] have tested object knowledge in a group of patients with MCI, AD and healthy seniors. The MCI group was impaired on all three conditions of the semantic object knowledge battery when compared to the control group, suggesting that semantic memory is impaired in MCI. Recent studies have also suggested that MCI patients may suffer from early breakdown of famous people knowledge [8, 21]. In the study by Vogel et al. [21], the aim was to determine if semantic impairments could be detect-

ISSN 0953-4180/08/$17.00 © 2008 – IOS Press and the authors. All rights reserved ed in predementia AD. Using a number of short neuropsychological tests assessing semantic memory, the authors demonstrated that category fluency and naming famous faces were the most frequently impaired tests in predementia patients who later on converted to AD. Estevez-Gonzalez et al. [8] also examined whether impaired naming of famous faces specifically, could be found in the preclinical phase of the disease. Results showed that MCI patients who later on converted to AD performed significantly worse on this task than the normal control group, while the group of MCI patients who did not convert to AD at a 2-year follow-up obtained intermediate results between the normal control group and the group of MCI patients who converted.

In summary, the literature seems to point to different aspects of semantic memory that appear to be affected in MCI. Nonetheless, it is not clear whether certain categories of conceptual knowledge are affected more than others and this question has never been addressed directly. This leads us to question whether MCI patients have deficits limited to one domain of conceptual knowledge or whether impairments are found across multiple domains of semantic memory. The aims of the present study were thus (i) to examine whether MCI patients present with semantic deficits; (ii) to examine the performance of a group of amnestic MCI patients on tasks that require identifying common objects, famous people and famous public events. Our hypothesis was that knowledge about famous people and famous public events will be more affected than object knowledge because the former have more distinctive and unique features which make them more vulnerable to brain damage. A final objective of this cross-sectional investigation was to examine the proportion of patients with MCI who present with semantic deficits.

## 2. Methods

### 2.1. Subjects

Two groups of French-speaking participants were included in the study. The amnestic MCI group consisted of elderly subjects referred to the Neurology and Neuropsychology Unit at the Timone Hospital for memory complaint. Criteria for amnestic MCI correspond to the classic criteria [17] and include: intact activities of daily living such as assessed by an interview and an IADL score $= 0$ [15]; MMSE score $\geqslant 25$ [9]; a subjective memory complaint; an objective and predominant memory impairment $\leqslant -1.5$ SD to the healthy population; absence of dementia. Twenty patients meeting these criteria were included in this study. In addition, these patients did not present with systemic disease, neurological or psychiatric disorder. 20 matched control subjects with no neurological or psychiatric disturbances were also included in the study. Controls were matched to MCI participants for age and level of education (number of years of formal education). All participants and their families gave informed consent before participation and participated to the study in accord with institutional guidelines.

Table 1
Summary of demographic data and MMSE scores

| General | MCI | SD | Controls | SD |
|---|---|---|---|---|
| Age | 73.3 | 7.1 | 73.2 | 11.4 |
| Education (years) | 11.1 | 5.3 | 10.8 | 3.7 |
| MMSE (30) | 26.8 | 2.4 | 29.1 | 1.2 |

### 2.2. General neuropsychology

All patients underwent a general neuropsychological evaluation in order to provide a global measure of their cognitive functioning in addition to their neurological examination. The following battery of neuropsychological tests was administered: the MMSE as a general measure of cognitive impairment [9]; executive functions were assessed with the Trail Making Test (parts A and B), verbal fluency for the letter P and category fluency (animals) in two minutes and the Matrix Design subtest of the WAIS-III [22]; language was assessed using the DO80 picture naming test [6]; visuoperceptual functions were assessed with the Benton Facial Recognition Test [3]; memory was assessed using the 5 words test [7], the digit span subtest [23] and the Matrix Design subtest of the WAIS-III [22]. Results of the demographic data and of the neuropsychological evaluation are presented in Tables 1 and 2, respectively.

### 2.3. The semantic memory battery

This battery was devised locally in Marseille by S.J. It comprises different subtests, but for the purpose of the present study only the following subtests were used:

#### 2.3.1. Test of objects

This part of the battery assesses general knowledge of common objects. The subtest consists of naming and identifying 20 photographs of objects including tools, animals, etc. The objects selected consisted mostly of low-frequency items in order to detect potential sub-

Table 2
Summary of the neuropsychological data of MCI participants. Normal scores are indicated in brackets

| | MCI | SD | Normal score |
|---|---|---|---|
| *Memory* | | | |
| 5 words test (immediate) | 4.5* | 0.8 | 5 (0) |
| 5 words test (delayed) | 3.5* | 1.5 | 5 (0) |
| Forward digit span | 5.2 | 0.9 | 6.5 (2.3) |
| Backward digit span | 3.8 | 1.0 | 5.9 (2.4) |
| *Language* | | | |
| DO80 | 78.3 | 2.4 | 77.8 (2.2) |
| *Executive functions* | | | |
| TMT A (sec) | 57.6 | 28.3 | 60 (26) |
| TMT B (sec) | 119 | 57 | 152 (83) |
| Letter fluency (letter P) | 22.8 | 8.9 | 23.8 (8.4) |
| Category fluency (animals) | 17.2 | 6.9 | 32.6 (7.9) |
| Matrix reasoning (WAIS-III) | 9.9 | 2.3 | 7 (3) |
| *Visuospatial abilities* | | | |
| Benton Facial Recognition Test | 46.3 | 3.8 | 41–54 |

*Indicates pathological scores ($<$ 2 standard deviations).

tle impairments that could not be detected using more frequent items found in more commonly used tests (e.g. the DO80). Participants were given one point if they were able to name the object upon presentation of the photograph and one point if they were able to provide information about the object. Mean scores for each participant were converted in a percentage score reflecting the accuracy of overall performance.

### 2.3.2. *Test of famous people*

This part of the battery consists of naming and providing general information about famous people from their photographs. 10 photographs of famous people (actors, singers, politicians, professional athletes, etc.) are presented one by one, and the subject is instructed to name and to provide as much specific biographical information as possible about the famous person. Famous people include both recent celebrities (from the past 10 years) and older celebrities (from the past 10 to 50 years). Again, participants were given one point if they were able to name the famous person upon presentation of the photograph and one point if they were able to provide specific semantic information about the person. Mean scores for each participant were converted in a percentage score reflecting the accuracy of overall performance.

### 2.3.3. *Test of famous public events*

This part of the battery consists of naming and providing general information about 10 famous public events from their photographs. They include photographs of recent (events that have occurred in the past 10 years) and more remote events (events from the past 10 to 50 years). Examples of famous events include Armstrong on the moon, Hiroshima, etc. Participants were given one point if they were able to name the famous event upon presentation of the photograph and one point if they were able to provide specific semantic information about the person. Mean scores for each participant were converted in a percentage score reflecting the accuracy of overall performance.

## 3. Statistical analysis and results

The data were analyzed using analyses of variance ($p < 0.05$). First, differences between the 2 groups in terms of MMSE score, age and education were analyzed using one-way analyses of variance (ANOVA). A two-way ANOVA was then carried out to compare group performance (expressed in terms of percentage correct response) on the different tests of the semantic battery.

The MCI and control groups did not differ on age ($F = 0.038$; $p = 0.832$). Importantly for the assessment of semantic memory, education level was identical in the two groups ($F = 0.042$; $p = 0.839$). MMSE scores were different between the two groups ($F = 12.51$; $p < 0.001$) as expected. Prior to performing an ANOVA, the normality of distribution of results for both groups was verified and confirmed by calculating skewness and kurtosis z-scores. A two-way analysis of variance (ANOVA) with one within factor, category (objects, famous persons, famous public events) and one between factor, group (control, MCI) was then carried out on percentage correct response. Analysis of performance (expressed in percentage) revealed significant main effects of semantic category ($F_{(2,76)} = 19.2$; $p < 0.001$) and group ($F_{(1,38)} = 18.2$; $p < 0.001$), as well as a significant semantic category x group interaction ($F_{(2,76)} = 7.49$; $p < 0.001$). Overall, the MCI group showed significantly better performance on the Test of objects than on the Test of famous people and on the Test of famous events, respectively ($p < 0.001$) (see Fig. 1). Effects on semantic tests reflected failure to provide specific semantic information about persons or events. Finally, a plot of the performance of individual MCI subjects on the Test of famous people is presented in Fig. 2. As can be observed, half of the MCI patients perform within the normal range, while the other half perform more than two standard deviations below the control subjects.

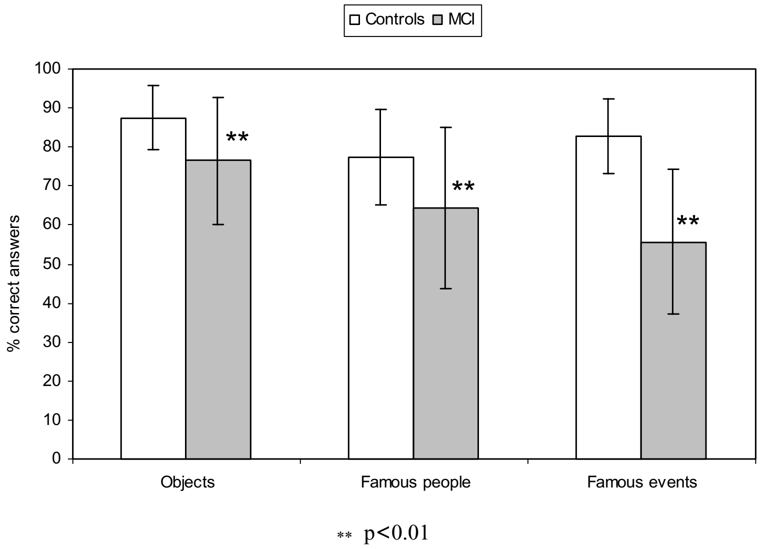

Fig. 1. Performance of the MCI and control groups on the different tests of the semantic battery. Results indicate significant differences between controls and MCI patients on all three subtests. The difference between the groups is more pronounced on the Famous public events test and on the Test of famous people, respectively, than on the Test of objects.

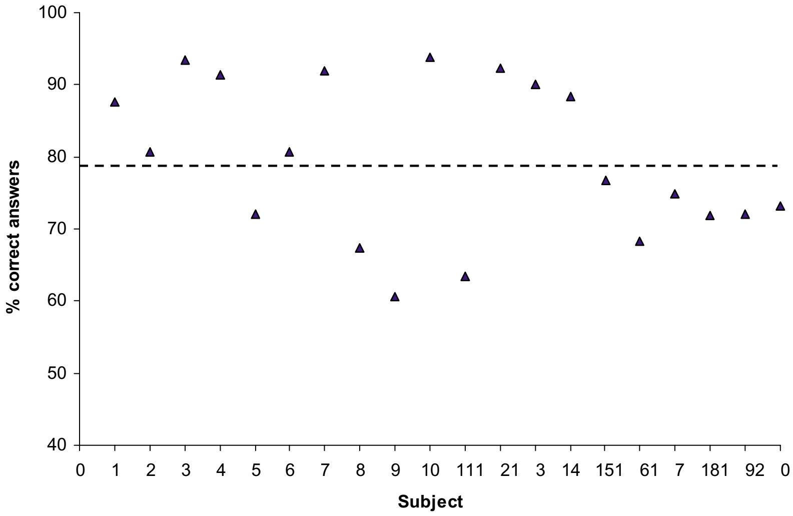

Fig. 2. Individual performance of MCI participants on the Test of famous persons. The dotted line indicates normal cut-off score (performance under that cut-off represents less than two standard deviations below normal controls). Results indicate that 50% of the MCI participants perform under the cut-off score.

## 4. Discussion

Our results, in agreement with recent studies, point to a clear semantic deficit in amnestic MCI patients. The latter were impaired on each of the three clinical tests of the semantic battery employed in this study. All tests were presented in the visual modality (from photographs) and were thus comparable on this dimension. Performance on the Benton Facial Recognition Test was normal and ensured that the impairment of the MCI patients was not related to visuoperceptual dysfunctions. Likewise, performance on the Matrix reasoning subtest, a test of executive functioning based on visual material, was also normal suggesting that the poor performance of MCI patients could not be related to difficulties in processing complex visual material. These results confirm the view that more detailed neuropsychological testing may help isolate semantic defects in MCI that are difficult to detect using more conventional neuropsychological tests.

The fact that the MCI group was impaired on all three of the semantic subtests has several implications. First, MCI performance appears to be independent of task difficulty. It could have been argued that photographs of famous events are simply more difficult than photographs of objects. However, performance of the control group was quite similar across the three semantic tests while performance of the MCI group was statistically more impaired on the Test of famous events and the Test of famous people, respectively, than on the Test of objects (see Fig. 1). In addition, the MCI group and the control group obtained respectively 77% and 63% correct answers on the Test of famous people, and 83% and 56% on the Test of famous events. Thus, although performance of the control group was better on the Test of famous events than on the Test of famous people, the reverse pattern of performance was observe for the MCI group. This result seems to argue against the idea that differences in performance across tasks in the MCI population reflect differences in task difficulty. The fact that performance was independent of task difficulty supports the notion of a genuine semantic impairment in the MCI population.

Why do MCI patients have semantic deficits? There are two possible neuroanatomical interpretations. The first explanation is that preclinical AD patients suffer from lesions of the subhippocampal area in the very early stage before the hippocampus is affected [4], and that this region may be involved predominantly in context-free, item-specific memory and semantic memory [2, 16,20]. Early neuropathological lesions to the perirhinal cortex and adjacent entorhinal cortex should thus yield semantic disturbances in preclinical AD. According to a different view, episodic memory and semantic memory are both dependant on the integrity of medial temporal lobe structures, and lesions to this region should cause equal impairments in episodic and semantic memory [18]. Consequently, semantic impairments could thus be considered to be a "normal" consequence of medial temporal lobe pathology in the clinical presentation of AD. Currently, both of these interpretations are consistent with the few reports in the literature as well as with our own results suggesting that preclinical AD patients exhibit semantic deficits.

Furthermore, why do MCI patients exhibit greater difficulties identifying famous people and famous events than common objects? This study is the first to our knowledge to compare directly different domains of semantic memory in MCI and to clearly demonstrate differential impairment in these domains. Estevez-Gonzalez et al. [8] suggested that specific neural circuitry within the temporal lobe involved in the recognition and identification of famous people may be affected early in the disease process. Although person-based semantic knowledge can be affected predominantly in other neurodegenerative conditions [14], our present results do not support this view within the context of MCI, since knowledge about famous events was affected to a similar extent than person-based knowledge. We suggest that entities such as famous people and famous events are unique in the sense that they contain many idiosyncratic attributes that are specific to each one of them and whose properties are not shared by other members of the same category. In contrast, common objects are characterized by more generic, non-unique features that can be shared by many members of a same category. It is possible that the many distinctive and unique features that characterize famous people and famous public events may be more prone to breakdown and brain damage than concepts such as common objects sharing greater interconnected features with similar concepts of a same category.

A last point that deserves attention in this study is the data presented in Fig. 2. Half of the MCI patients performed less than 2 standard deviations below controls, while the other half performed within the normal range. Thus, not all MCI patients present with semantic deficits. In contrast, episodic memory impairments are characteristic of all patients and are a core underlying feature of amnestic MCI [17]. Consequently, episodic memory impairments cannot help to discriminate between MCI patients who will later on convert to AD from those who will remain stable over the years. Although the results of this study are cross-sectional in nature and do not allow to make clear inferences about the fate of the patients, further studies are clearly needed to determine whether semantic memory impairments may help to predict longitudinally the risk of developing full-blown AD. It is possible that amnestic MCI patients with semantic deficits are those at greater risk of developing AD in the following years. In conclusion, semantic impairments were found in a group of amnestic MCI patients, despite normal performance on a range of more classic neuropsychological tests including picture naming and fluency tests. It is possible that specific semantic tests, when used together with other neuropsychological measures, may help improve early diagnosis of AD.

## Acknowledgements

Sven Joubert is supported by the Fonds de Recherche en Santé du Québec (FRSQ) and by the Alzheimer Society of Canada.
